# Correlative study between C-reactive protein, clinical severity, and nerve conduction studies in Guillain-Barrè syndrome

**DOI:** 10.1186/s41983-018-0006-2

**Published:** 2018-04-25

**Authors:** Yosria A. Altaweel, Sawsan Abdelaziz, Hala A. Fathy, Shimaa AbdelBadea

**Affiliations:** Neurology Department, Faculty of Medicine, Zagazig University, Zagazig, Egypt

**Keywords:** GBS, CRP level, Nerve conduction studies, Prognosis

## Abstract

**Background:**

Guillain-Barre' syndrome (GBS) is a serious autoimmune disorder in which the immune system attacks healthy nerve cells of the peripheral nervous system causing polyradiculoneuropathy which leads to weakness, numbness, and tingling, and can eventually cause paralysis. Autoimmune conditions like GBS can induce a high level of inflammation resulting in an increase in the C-reactive protein( CRP) production. The aim of this study is to assess the relationship between CRP level and the clinical severity as well as the electrophysiological findings of nerve conduction studies in patients with GBS.

**Methods:**

Twenty- four patients (10 males &14 females) with ages ranged from 14 to 50 years and a mean age of 33.46 ±12.25 years who fulfilled the clinical criteria for diagnosing GBS were recruited within the first 2 weeks of onset of illness, in a cross- section study. They underwent general and neurological examination. Nerve conduction studies as well as assessment of serum CRP level were done.

**Results:**

There was a statistically significant positive correlation between clinical severity assessed by (Hughes disability scale) and serum CRP level in GBS patients. Multivariate logistic regression analysis showed that both gastroenteritis, cranio-bulbar affection, need for mechanical ventilation (MV), disability score >4, and absent motor and sensory responses were significantly associated with high serum CRP level >6mg/dl.

**Conclusions:**

The results of this study support the hypothesis that in GBS patients, gastroenteritis, craniobulbar affection, need for MV, disability score >4, and absent motor and sensory nerve responses were significantly related to high serum CRP level. This reflects the negative impact of the inflammatory response elicited by high CRP level on clinical severity in GBS patients, and so it may be used as a prognostic marker of clinical severity of GBS and this can help in therapeutic decision making.

## Background

Guillain-Barré syndrome (GBS) is a severe autoimmune disorder in which the immune system forms auto-antibodies against healthy nerve cells of the peripheral nervous system causing polyradiculoneuropathy which in turn causes weakness, numbness, and tingling and can eventually cause paralysis. The cause of this condition is unknown, but it is typically triggered by many infectious illnesses like gastroenteritis and respiratory tract infection (Yuki and Hartung 2012). It is generally precipitated 1–3 weeks following many infections like respiratory tract infection and *Campylobacter jejuni* infection which suggest an underlying humoral immunopathogenic mechanism (Yu et al. 2006).

C-reactive protein (CRP) is an acute phase reactant produced by the liver in response to factors released by macrophages and adipocytes. Its level rises dramatically during inflammatory processes occurring in the body, and it is a critical component of the immune response (Lau et al. 2005), Moreover, CRP plays an important role in innate immunity as an early defense system against infections and its level rises when there is a microbial infection or an inflammation without microbes (Peaceman et al. 1997). Autoimmune conditions like GBS can stimulate the production of a high level of inflammation resulting in an increase in the CRP production, so highly sensitive CRP assay may become a new risk assessment marker in the future for autoimmune disorders including GBS (Vaishnavi et al. 2014). In addition, measuring CRP levels can help to screen both infections and inflammatory diseases; also, dynamic serial measurement of CRP has been widely used to help therapeutic decision making (Lobo 2012).

Electrophysiology including nerve conduction study (NCS) plays a determinant role in GBS diagnosis and classification of the subtypes and in establishing prognosis (Uncini and Kuwabara 2012). Diagnostic sets have been proposed for assessment of acute inflammatory demyelinating neuropathy (AIDP), acute motor axonal neuropathy (AMAN), and acute motor and sensory axonal neuropathy (AMSAN). Nerve conduction studies should be repeated for proper diagnosis of GBS subtypes, identification of pathophysiological mechanisms, and hence assessment of prognosis (Uncini and Kuwabara 2012).

### Aim of the study

This study was done to assess the relation between serum CRP level and the clinical severity as well as the electrophysiological findings of nerve conduction studies in patients with GBS.

## Methods

A cross-sectional study was conducted in the Neurology Department, Zagazig University Hospitals during the period from February 2015 to July 2016. Twenty-four patients (10 males, 14 females) which fulfilled the clinical criteria for diagnosis of GBS according to Asbury and Cornblath (1990) were recruited in the study. Their ages ranged from 14 to 50 years with a mean age 33.46 ± 12.25 years.

All patients in the study had fulfilled the following inclusion and exclusion criteria:

1. Inclusion criteria:

Patients with diagnosis of GBS based on criteria of Asbury and Corrblath (1990) within the first 2 weeks following illness onset were included in our study.

- Features required for diagnosis:
  * Progressive weakness in both arms and legs (might start with weakness only in the legs)
  * Areflexia (or decreased tendon reflexes)
- Features that strongly support diagnosis:
  * Progression of symptoms over days to 4 weeks
  * Relative symmetry of symptoms
  * Mild sensory symptoms or signs
  * Cranial nerve involvement, especially bilateral weakness of facial muscles

2. Exclusion criteria:

- Any other neurological illness whether acute or chronic
- Any other causes which can affect NCSs, e.g., drugs, toxins, diabetes mellitus (DM)
- Concomitant inflammatory disease whether infectious or non-infectious, within the last 2 weeks
- Tumors, metastases, and immunomodulatory therapy within the last 6 months
- History of surgery or significant trauma within the last 2 weeks
- Patients with system failure (respiratory, hepatic, renal, cardiac)
- Pregnant females
- Severe psychiatric illness

**Table:** 

0	A healthy state
1	Minor symptoms and capable of running
2	Able to walk 10 m or more without assistance but unable to run
3	Able to walk 10 m across an open space with help
4	Bedridden or chair bound
5	Requiring ventilation for at least part of the day
6	Dead

All patients in this study were subjected to the following:

1. Complete history taking stressing on the onset, course, and duration of the disease. Also, history of previous gastroenteritis or upper respiratory tract infection
2. Complete general examination
3. Neurological examination including assessment of clinical GBS severity using a disability scale adapted from Hughes and Rees (1997)

GBS disability scale (Hughes and Rees 1997)

4. ECG to detect any concomitant arrhythmias
5. Laboratory investigations include the following:

- Routine investigations:
  * Complete blood count (CBC)
  * Erythrocyte sedimentation rate (ESR)
  * Blood glucose level
  * Liver function tests (LFT) and kidney function test (KFT)
- Quantitative assessment of serum CRP using latex: within 2 weeks of symptom onset. CRP-Turbilatex is a quantitative turbidimetric test for the measurement of CRP in human serum or plasma. Latex particles coated with specific human anti-CRP are agglutinated when mixed with samples containing CRP. The agglutination causes an absorbance change, dependent upon the CRP contents of the patient sample that can be quantified by comparison from a calibrator of known CRP concentration. Normal concentration in healthy human serum is usually lower than 10 mg/l, slightly increasing with aging. Higher levels are found in late pregnant women, mild inflammation and viral infections (10–40 mg/l), active inflammation, bacterial infection (40–200 mg/l), severe bacterial infection, and burns (> 200 mg/l) (Clyne and Olshaker 1999).

6. Nerve conduction study:

Nerve conduction studies (NCSs) were done with an EMG machine (Nemus, Biomedica, Model number 00655, Galileo NT software version 3.71/00, Italy) within 2 weeks of onset of symptoms.

All patients in our study had NCSs in at least four motor nerves according to the following protocol (Gordon and Willbourn 2001):

* Motor NCS for median, ulnar, common peroneal, and posterior tibial nerves
* Sensory NCS for median, ulnar, sural, and superficial peroneal nerves were recorded antidromically with surface ring (median and ulnar nerves) and disc (sural and superficial peroneal nerves) electrodes
* Late response: F-waves were recorded following at least 10 distal stimuli to determine minimal F-wave latency or F-wave absence

The following parameters were measured:

* Distal latency (DL), compound motor action potential (CMAP) amplitude, and conduction velocity (CV) of motor and sensory nerves
* Peak sensory latency, sensory nerve action potential amplitude (SNAP)
* F-wave (latency and persistence)

Patients were classified as having:

1. *Demyelinating polyradiculoneuropathy* consistent with GBS if there were a combination of prolonged distal motor latency (> 150% of the upper limit of normal), CV (< 70% of the lower limit of normal), and prolongation of F-wave latency (> 150% of the upper limit of normal)
2. *Axonal polyradiculoneuropathy* if there was low CMAP amplitude, normal conduction velocity or slightly slowed, and normal distal latency or slightly prolonged

The values for each variable were compared with the standard ranges of normality.

### Ethical consideration

This is the grant number for ethics approval (IRB#: 1879-15-2-2015). A written informed consent was obtained from every patient or his/her relative to be included in the study. This study was approved by the institute research board of Faculty of Medicine, Zagazig University.

### Statistical analysis

The obtained data were tabulated and analyzed using Statistical Package of Social Science (SPSS version, 22) (Levesque 2007). Continuous variables were expressed as mean ± SD (standard deviation) and median. The means were compared with independent Student’s *t* test. Categorical variables were compared using the chi-square test. Correlation coefficient (Spearman’s or Pearson’s) was calculated to assess the relation between serum CRP levels and clinical severity of GBS using Hughes disability scale. Odds ratio (ORs) and confidence intervals (CIs) were also calculated in a logistic regression model to assess the independent factors that affect prognosis of GBS. A difference was considered statistically significant if *P* value was ≤ 0.05.

## Results

A total of 24 GBS cases with ages ranged from 14 to 50 years (mean age 33.46 ± 12.25 years) were included in this cross-sectional study according to the above mentioned inclusion and exclusion criteria.

Female patients represented 58.3% and male patients represented 41.7% with ages ranged from 14 to 50 years with mean age of 33.46 ± 12.25 years. The most common preceding infection was gastroenteritis (41.7%) while upper respiratory tract infection (URTI) presented only 25%. All the patients were presented by both motor and sensory symptoms. The most common cranial nerve involved was facial (54.2%), followed by bulbar cranial nerves (25%). 16.7% of patients needed MV (Table 1).

**Table 1:** Demographic data and clinical presentation in the studied GBS cases

Variables	(*N* = 24)	
Age (years)		
Range	14–50	
Mean ± SD	33.46 ± 12.25	
	*N*	%
Sex		
Male	10	41.7
Female	14	58.3
Preceding infection		
No	8	33.3
Gastroenteritis	10	41.7
URTI	6	25
Clinical presentation		
Motor symptoms	24	100
Sensory symptoms	24	100
Cranial nerve involvement		
Facial	13	54.2
Bulbar	6	25
Autonomic symptoms	4	16.7
MV	4	16.7

*N* number, *MV* mechanical ventilator, *URTI* upper respiratory tract infection

The level of CRP in our patients ranged from 0.4 to 20.5 mg/dl with mean serum level of 5.52 ± 4.57 mg/dl; patients with CRP level ≤ 6 mg/dl represented 66.7%, while those with CRP levels > 6 mg/dl were 33.5% (Table 2).

**Table 2:** Mean serum CRP level (mg/dl) in the studied GBS cases

Mean serum CRP level ± (SD)	(*N* = 24)	
CRP (mg/dl)		
Mean ± SD	5.52 ± 4.57	
Range	0.4–20.5	
Cutoff point of CRP level	N	%
≤ 6 mg/dl	16	66.7
> 6 mg/dl	8	33.3

*CRP* C-reactive protein, *N* number, % percentage

The electrophysiological findings of NCSs in the studied GBS patients showed that mixed axonal and demyelinating polyneuropathy was the most frequent type as it occurred in 45.8% of cases, whereas demyelinating type was found in 29.2% of cases, and axonal in 8.3% of cases (Table 3).

**Table 3:** Types of electrophysiological findings of the studied GBS cases

Variable	(*N* = 24)	
	*N*	%
Electrophysiological finding		
Normal	4	16.7
Demyelinating	7	29.2
Axonal	2	8.3
Mixed	11	45.8

Motor and sensory nerve conduction study findings among studied GBS cases showed that absent motor response was found in 20.8% of cases, and low CMAP amplitude in 54.2%. F-wave abnormality was more common (70.8%), then prolonged distal and motor latency (66.7%) and slowing of CV (37.5%). As regards sensory nerve conduction study findings, prolonged latency and low SNAP amplitude were the most prominent features (16.7%), whereas absent sensory response was found in 8.3% of cases (Table 4).

**Table 4:** Motor and sensory nerve conduction study findings in GBS cases

Variable	(*N* = 24)	
	*N*	%
Motor nerve conduction study		
Absent motor response	5	20.8
Slow CV	9	37.5
Prolonged distal latency	16	66.7
Low CMAP amplitude	13	54.2
F-wave abnormality	17	70.8
Sensory nerve conduction study		
Prolonged latency	4	16.7
Absent response	2	8.3
Reduced SNAP amplitude	4	16.7

Abnormality means finding in ≥ 2 nerves

*CV* conduction velocity, *CMAP* compound motor action potential, *SNAP* sensory-neural action potential

The relation between Hughes disability scale score and mean serum CRP level among the studied GBS cases demonstrates that there was statistically significant relation between serum CRP and disability scale score (*p* = 0.01) as the higher the CRP level, the worse the disability score of the patients (Table 5).

**Table 5:** Relation between Hughes disability scale score and mean serum CRP level among the studied GBS patients

Disability score (Hughes disability scale)	*N*	CRP (mg/dl) X ± SD	K	*P*
Grade				
2	3	3.33 ± 1.74	3.28	0.01*
3	1	4.4		
4	15	5.75 ± 3.58		
5	3	8.45 ± 5.3		
6	2	11.3 ± 8		

*CRP* C-reactive protein

*: significant

By multivariate logistic regression analysis, it was found that both preceding infection with gastroenteritis, craniobulbar affection, need for MV, disability score > 4, and absent motor and sensory nerve responses were significantly related to high serum CRP level > 6 mg/dl (Table 6).

**Table 6:** Multivariate regression analysis for significant findings in GBS patients with serum CRP > 6 mg/dl

Variable	B	SE	Wald	Sig.	Exp(B)	95.0% CI	
						Lower	Upper
Had GE	1.82	0.56	7.12	0.04*	3.45	2.12	7.14
Positive craniobulbar manifestation	0.81	0.43	0.53	0.61	0.79	0.354	1.791
Need for MV	3.12	0.54	11.22	< 0.001**	9.56	4.56	16.34
Disability score > 4	3.83	0.64	5.98	0.03*	2.98	1.85	4.34
Absent motor response	1.67	0.45	7.88	0.02*	2.38	1.98	6.55
Low CMAP amplitude	−0.26	0.39	0.43	0.76	0.85	0.353 0.48	0.353 0.99
Absent sensory response	−1.56	0.54	7.45	0.01*	2.76	1.87	7.86

*CRP* C-reactive protein, *B* the estimated logit coefficient, *SE* standard error, *CI* confidence interval, *GE* gastroenteritis, *MV* mechanical ventilator, *CMAP* compound motor action potential, *Sig.* significance

*: significat

**: highly significant

## Discussion

Guillain-Barré syndrome is a clinically diagnosed disorder, but nerve conduction studies can help to support the diagnosis and discriminate between axonal and demyelinating subtypes and could relate to prognosis (Chiò et al. 2003). C-reactive protein (CRP) is one of acute phase proteins, the serum or plasma levels of which rise during general non-specific response to a wide variety of diseases, either infectious or inflammatory including autoimmune diseases (Lau et al. 2005).

Several studies agree that GBS can occur at any age (Hughes and Rees 1997). In the present study, the mean age of GBS patients was 33.46 (±12.25) years. Our results were in accordance with that obtained by Mohamed et al. (2016), where the mean age of their patients was 38.74 years. However, a higher mean age of 43.7 years was reported by González-Suárez et al. (2013).

Concerning sex distribution, it was reported that GBS was more frequent in females (58.3% females vs 41.7% males) in our study. Other studies found that GBS was more common in male patients (Hughes and Cornblath 2005; Sejvar et al. 2011; Dhadke et al. 2013; Fokke et al. 2014). However, González-Suárezand et al. (2013) in their study found no significant difference between both genders among GBS patients.

Regarding preceding infections in our study, it was found that about 41.7% had gastroenteritis and 25% had URTI while only 33.3% had no history of any infection prior to presentation. These data were in agreement with results obtained by Newswanger and Warren (2004) who identified *C. jejuni* as the most frequent antecedent pathogen of GBS. Sudulguntaand et al. (2015) in their study found that the most common antecedent events associated with GBS was gastrointestinal infection (47.25%) followed by fever and cough which represented 42.62%, while upper respiratory tract infection was reported to be the most common preceding infection in studies done by Chiò et al. (2003) and Willison (2005).

Patients with Guillain-Barré syndrome differ from each other regarding the degree as well as distribution of motor and sensory symptoms plus the presence of cranial nerve deficits, autonomic dysfunction, and preceding infection. Regarding the clinical presentation, we found that all patients (100%) had both motor and sensory symptoms, while 54.2% had facial nerve involvement, 16.7% had autonomic symptoms, and 16.7% needed MV. Fokke et al. (2014) and Kalita and Misra (2014) reported that motor weakness was the most frequent symptom (73.8–100%) and sensory symptoms represented 52.7% and cranial nerve affection represented 53–62.5%. Other studies (Dhadke et al. 2013; Fokke et al. 2014; Kalita and Misra 2014) found that dysautonomia represented 20.4% and MV was found in 13.1–28%. A low frequency of sensory manifestations (32.5%) was recorded by Dhadke et al. (2013).

The Hughes disability scale was used to assess clinical severity of GBS in our study. 16.7% of our patients were able to walk either without assistance or with help (grades 2 and 3). The majority of our patients (62.5%) were bedridden or chair bound (grade 4) and 12.5% needed mechanical ventilation (grade 5). This was in agreement with data obtained by Parmar et al. (2013) who reported that the majority of their GBS patients were in grade 4 (83.7%). The author explained that the reason for the high frequency of severe disability was the delay in seeking treatment.

Regarding the electrophysiological studies in our GBS patients, we found that mixed axonal and demyelinating polyneuropathy was the most common type (45.8%) followed by demyelinating polyneuropathy (29.2%) and axonal polyneuropathy (8.3%). In accordance with our result, Walling and Dickson (2013) found that multifocal demyelinating polyneuropathy with secondary axonal degeneration was the most common subtype. However, other studies found that demyelinating polyneuropathy was the predominant subtype followed by demyelinating and axonal polyneuropathy (Fokke et al. 2014; Parmar et al. 2013). This variability of subtypes of GBS among countries could be due to different genetic background and environmental exposures (Parmar et al. 2013).

As regards electrophysiological findings of NCSs among our patients, it was also found that F-wave abnormality was the most common finding (70.8%), then prolonged distal latency (DL) (66.7%), low compound motor action potential (CMAP) amplitude (54.2%), and slow conduction velocity (37.5%), and the least was absent motor response (20%). Whereas in sensory nerve conduction studies, prolonged latency as well as reduced SNAP amplitude were the most prominent finding (16.7%). Our results were in agreement with that of Parmar et al. (2013) who found that the most common electrodiagnostic abnormalities were absent or prolonged F-wave (90%), prolonged DL (80%), delayed CV (73%), partial or complete conduction block (63%), reduced CMAP amplitude (38%), and abnormal SNAP (28%). The authors explained the predominance of F-wave abnormalities in GBS patients by an axonal dysfunction in proximal nerve segment or by axonal degeneration at the level of nerve root (Parmar et al. 2013).

C-reactive protein is one of the acute-phase proteins, which rises during a wide range of acute and chronic inflammation (Vaishnavi et al. 2014). Also, CRP has a role in autoimmune diseases as it can bind to auto-antigens; thus, immune-mediated conditions like GBS can stimulate inflammatory response which in turn lead to in an increase in CRP level (Szalai 2004). In the present study, CRP level > 6 mg/dl were found in 33.5% of our patients. This was in agreement with Vaishnavi et al. (2014) who found that 24.4% of their patients were positive for CRP. Although there were few studies concerning CRP serum levels in patients with GBS, a high level of CRP was reported in other studies (Daniel 2011; Chen et al. 2013).

On doing a multivariate regression analysis, it was found that serum CRP level > 6 mg/dl was significantly associated with preceding infection (gastroenteritis), need for MV, disability score > 4, and absent both motor and sensory nerve responses. Our results were in agreement with the study done by Rajabally and Uncini (2012) who found that predictors of bad prognosis were higher GBS disability score at 2 weeks, preceding diarrheal illness, faciobulbar palsy, need for MV, low CMAP amplitude, and absent motor responses. Also, Ortiz-Corredor et al. (2007) studied motor recovery after GBS in childhood, using univariate analysis and found that cranial nerve impairment, requirement of assisted ventilation, presence of quadriplegia, and presence of non-excitable motor nerves were associated with delayed motor recovery time. In explanation for that significant association, Vaishnavi et al. (2014) postulated that autoimmune conditions like GBS can stimulate inflammatory response and hence result in an increase in CRP production which in turn significantly related to clinical severity and severe disability resulting in poor prognosis in GBS patients.

## Conclusions

The results of the current study support the hypothesis that in GBS patients, history of preceding gastroenteritis, craniobulbar affection, need for MV, disability score > 4, and absent motor and sensory nerve responses were significantly associated with high serum CRP level, reflecting the negative impact of the inflammatory response on clinical severity and prognosis. Serial measurement of CRP can be used as risk assessment and prognostic marker.

### Study limitations

The study had some limitations as small sample size, and the study was a cross-sectional one.
